# Hydrogen Sulfide Stimulates Ischemic Vascular Remodeling Through Nitric Oxide Synthase and Nitrite Reduction Activity Regulating Hypoxia‐Inducible Factor‐1α and Vascular Endothelial Growth Factor–Dependent Angiogenesis

**DOI:** 10.1161/JAHA.112.004093

**Published:** 2012-10-25

**Authors:** Shyamal C. Bir, Gopi K. Kolluru, Paul McCarthy, Xinggui Shen, Sibile Pardue, Christopher B. Pattillo, Christopher G. Kevil

**Affiliations:** Departments of Pathology and Medicine, LSU Health Sciences Center–Shreveport, Shreveport, LA

**Keywords:** angiogenesis, ischemia, nitric oxide, vascular endothelial growth factor, xanthine oxidase

## Abstract

**Background:**

Hydrogen sulfide (H_2_S) therapy is recognized as a modulator of vascular function during tissue ischemia with the notion of potential interactions of nitric oxide (NO) metabolism. However, little is known about specific biochemical mechanisms or the importance of H_2_S activation of NO metabolism during ischemic tissue vascular remodeling. The goal of this study was to determine the effect of H_2_S on NO metabolism during chronic tissue ischemia and subsequent effects on ischemic vascular remodeling responses.

**Methods and Results:**

The unilateral, permanent femoral artery ligation model of hind‐limb ischemia was performed in C57BL/6J wild‐type and endothelial NO synthase–knockout mice to evaluate exogenous H_2_S effects on NO bioavailability and ischemic revascularization. We found that H_2_S selectively restored chronic ischemic tissue function and viability by enhancing NO production involving both endothelial NO synthase and nitrite reduction mechanisms. Importantly, H_2_S increased ischemic tissue xanthine oxidase activity, hind‐limb blood flow, and angiogenesis, which were blunted by the xanthine oxidase inhibitor febuxostat. H_2_S treatment increased ischemic tissue and endothelial cell hypoxia‐inducible factor‐1α expression and activity and vascular endothelial growth factor protein expression and function in a NO‐dependent manner that was required for ischemic vascular remodeling.

**Conclusions:**

These data demonstrate that H_2_S differentially regulates NO metabolism during chronic tissue ischemia, highlighting novel biochemical pathways to increase NO bioavailability for ischemic vascular remodeling.

## Introduction

Hydrogen sulfide (H_2_S) is a colorless, flammable, water‐soluble gas with a pungent odor of rotten eggs,^1,2^ which is produced from various sources in mammalian tissues, where it is generated during cysteine/homocysteine metabolism.^3^ H_2_S is produced predominantly by tissue‐specific enzymes,^4,5^ including cystathionine‐β‐synthase, cystathionine‐γ‐lyase, and 3‐mercaptosulfurtransferase. Similar to other gaseous mediators (eg, nitric oxide [NO] and carbon monoxide), H_2_S plays an important role in cardiovascular, neuronal, and endocrine systems, including ischemic and inflammatory processes.^6–10^

H_2_S has been reported to be beneficial for cardiovascular dysfunctional states involving different responses. For example, H_2_S promotes vascular smooth muscle relaxation and induces vasodilation of isolated blood vessels by opening adenosine triphosphate–sensitive potassium (K‐ATP) channel.^11^ It has been reported that H_2_S potentiates antioxidant defenses via enhanced production of glutathione.^12^ H_2_S also promotes tissue cytoprotection, as illustrated by inhibition of myocyte apoptosis during myocardial ischemia‐reperfusion.^13^ More recently, it has been proposed that H_2_S–NO interactions could occur, yet clear insight into this relationship is lacking. Conflicting reports have shown that H_2_S can (1) directly inhibit NO synthase (NOS) enzyme activity, (2) confer protection against experimental cardiac arrest in an endothelial NOS (eNOS)–dependent manner, (3) interact with NO to form unique reactive species involved in cardiovascular function, and (4) inhibit phosphodiesterase‐5 activity potentiating NOS activity.^14–18^ Unfortunately, no mechanistic information exists about how H_2_S affects NO metabolism and bioavailability during ischemic tissue vascular remodeling.

Here, we identify novel H_2_S–NO crosstalk pathways, whereby H_2_S stimulates NOS expression as well as xanthine oxidoreductase (XO)–mediated nitrite anion reduction to NO, which subsequently regulates ischemic vascular remodeling responses. We also report measurement of therapeutic plasma and tissue free H_2_S levels and downstream molecular pathways that regulate these responses. These data demonstrate that H_2_S serves an important role in maintaining tissue NO bioavailability during chronic ischemia.

## Methods

### Chemicals and Reagents

General chemicals and tissue culture reagents were obtained from Sigma Chemicals. Anhydrous sodium sulfide was purchased from Alfa‐Aesar Inc. Anti‐Ki67 antibody was obtained from Abcam Inc (Cambridge, MA, USA). Anti‐CD31 antibody was obtained from BD Biosciences (San Jose, CA, USA). Vectashield plus DAPI was obtained from Vector Laboratories. All secondary fluorophore‐labeled antibodies were obtained from Jackson Immunoresearch Inc (West Grove, PA, USA). The murine MS‐1 endothelial cell line was obtained from ATCC.

### Animals and Experimental Procedures

#### Animals

C57BL/6J, B6.129P2‐*Nos3*^*tm1Unc*^/J (eNOS‐knockout) and Db/Db diabetic male mice were purchased from Jackson Labs and used in this study. Mice were maintained at the Association for Assessment and Accreditation of Laboratory Animal Care International–accredited Louisiana State University Health Science Center–Shreveport animal resource facility and were maintained in accordance with the National Research Council's *Guide for Care and Use of Laboratory Animals*. All animal studies were approved by the institutional animal care and use committee (protocol P‐08‐041) and conformed to the Guide for the Care and Use of Laboratory Animals published by the National Institutes of Health.

#### Mouse Hind‐Limb Ischemia Model and Treatment Routes

Hind‐limb ischemia was induced in 12‐ to 16‐week‐old C57Bl/6J wild‐type and eNOS‐knockout male mice and in 36‐week‐old Db/Db diabetic male mice, as we have reported previously.^19,20^ After anesthesia with ketamine/xylazine (100 mg/kg or 83 mg/kg) injection, ligation of the left femoral artery was performed. Mice were randomly assigned to different experimental groups by one investigator and were treated and evaluated by a second blinded investigator. Sodium sulfide with or without inhibitors was administered in the retro‐orbital capillary plexus.

#### Laser Doppler Blood Flow Measurements

Laser Doppler blood flows were measured with a Vasamedics Laserflo BPM2 device in the gastrocnemius before ligation, after ligation, and at the indicated days after ligation, as we have reported previously.^19,20^

#### Vascular Density and Cell Proliferation Measurements

Vascular density (anti‐CD31) and cell proliferation (anti‐Ki67) measurements were performed as we have reported previously.^19^ Briefly, the gastrocnemius muscles from ischemic and nonischemic hind limbs were removed, dissected, and embedded in Optimal Cutting Temperature freezing medium and were stained with anti‐CD31, anti‐Ki67, and DAPI for immunofluorescent imaging. Simple PCI software version 6.0 (Compix Inc, Sewickly, PA) was used to quantitatively measure the surface area of CD31, Ki67, and DAPI staining.

#### Vascular Endothelial Growth Factor Enzyme‐Linked Immunosorbent Assay

Ischemic and nonischemic tissue isolated from PBS control or H_2_S‐treated wild‐type/eNOS‐knockout mice was harvested at various time points, and vascular endothelial growth factor (VEGF) expression was measured by enzyme‐linked immunosorbent assay (ELISA) with the R&D VEGF Quantikine ELISA kit, as per the manufacturer's instructions.

#### Xanthine Oxidase Activity ELISA Assay

Ischemic and nonischemic tissue from control or H_2_S‐treated mice was harvested at day 10. Xanthine oxidase activity was measured with an ELISA kit from Cayman Chemicals, as per the manufacturer's instructions.

#### Cyclic Guanosine Monophosphate ELISA Assay

Ischemic and nonischemic tissue from control or H_2_S‐treated mice was harvested at day 10, and cyclic guanosine monophosphate (cGMP) level was measured with a cGMP assay from Sigma, as per the manufacturer's instructions.

#### Measurement of Plasma and Tissue Free H_2_S Levels

H_2_S was measured with monobromobimane (MBB) as we have reported.^21^ Thirty microliters of plasma or tissue supernatant was reacted with 70 μL of Tris‐HCl (100 mmol/L, pH 9.5, 0.1 mmol/L DTPA) and 50 μL of MBB solution (10 mmol/L) at 1% oxygen in a hypoxic chamber at room temperature. Sulfide‐dibimane (SDB) reaction product was analyzed with a Shimadzu Prominence Ultra Fast high‐performance liquid chromatography system. Specimen concentration was calculated by comparison to SDB sulfide standards.

#### Measurement of NO Generation

A Sievers 280i NO analyzer was used to measure H_2_S‐mediated NO production as we have reported.^19^ Muscle tissues were homogenized in Hank's balanced salt solution, and 200 μg total protein was injected into the reaction chamber for measurements of nitric oxides (NOx). Endothelial cells were exposed to 4 hours of either hypoxia (1% O_2_) or normoxia (21% O_2_) and were harvested, and 1×10^6^ cells in Hank's balanced salt solution were added into the reaction chamber. Sodium sulfide subsequently was injected into the chamber, and NO production was measured over time. Separate experiments with hypoxic endothelial cells plus the inhibitors 2‐4‐carboxyphenyl‐4,4,5,5‐tetramethylimidazoline‐1‐oxyl‐3‐oxide (cPTIO), paraformaldelhyde, sulfanilamide, N‐Ethylmaleimide (NEM), N5‐[imino(nitroamino)methyl]‐L‐ornithine, methyl ester (L‐NAME), and febuxostat also were performed. Cell free protein experiments with recombinant XO were added to the reaction chamber at concentrations of 0.01, 0.005, and 0.0025 units, and 50 μmol/L sodium sulfide was then injected into the chamber. NO production was determined by integrating the emission signal over time calibrated to a standard curve of nitrite (0.1, 0.5, 1, 10, and 100 μmol/L) reduced to NO in sodium iodide/glacial acetic acid.

### Statistical Analysis

Data were reported as mean±standard error of the mean for all groups. Statistical analysis was performed with Mann‐Whitney or Kruskal‐Wallis analysis of variance with Dunn's multiple‐comparison tests in GraphPad Prism software (GraphPad software, San Diego, CA).

## Results

### Plasma and Tissue H_2_S Levels Required for Ischemic Tissue Cytoprotection

**Figure 1.**
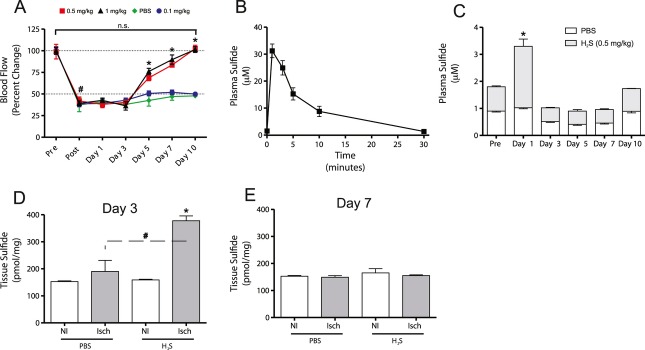
H_2_S restores blood flow in permanent femoral artery ligation–induced hind‐limb ischemia. A, Ischemic hind‐limb blood flow changes with increasing concentrations of sodium sulfide. B, Free plasma H_2_S levels after a single bolus injection of 0.5 mg/kg sodium sulfide. C, Steady‐state free plasma levels of H_2_S during the course of therapy. D, Tissue free H_2_S levels in ischemic (Isch) and nonischemic (NI) tissues at day 3 after ligation. E, Tissue free H_2_S levels in ischemic and nonischemic tissues at day 7 after ligation. n=12 animals per experimental cohort, *P<0.05 compared to control or nonischemic limb data; #*P*<0.05 ischemic limb comparison between PBS and H_2_S, or before and after ligation.

**Figure 2.**
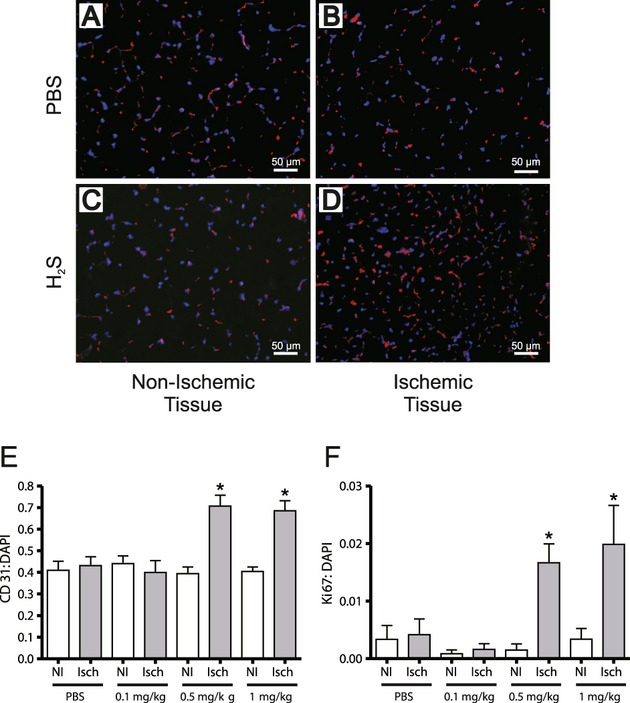
H_2_S enhances ischemic vascular density and cellular proliferation. A and B, Tissue sections from PBS‐treated animals both nonischemic and ischemic, respectively, stained for DAPI (blue) and CD31 (red) at day 10. C and D, Tissue sections from 0.5 mg/kg sodium sulfide–treated animals both nonischemic and ischemic, respectively, stained for DAPI (blue) and CD31 (red) at day 10. E, Graphical representation of the CD31:DAPI ratio (red:blue) demonstrating vascular density in different gastrocnemius tissues from sodium sulfide–treated mice. F, Graphical representation of Ki67:DAPI ratio (green:blue) demonstrating proliferation in different gastrocnemius tissues from sodium sulfide–treated mice. NI indicates nonischemic; Isch, ischemic. n=12 animals per cohort, *P<0.05 compared to PBS ischemic tissue data.

H_2_S has been reported to confer cytoprotection during various forms of tissue ischemia, yet little information is available about specific therapeutic concentrations of free H_2_S necessary for protective effects. Therefore, we examined a range of sodium sulfide doses (H_2_S donor), from 0.1 to 1 mg/kg, administered via retro‐orbital injection twice daily, to better understand therapeutic H_2_S levels necessary for ischemic tissue cytoprotection. Sodium sulfide doses of 0.5 mg/kg (12.8 μmol/kg per day) and 1 mg/kg (25.6 μmol/kg per day) significantly restored ischemic hind‐limb blood flow by day 10 after ligation (Figure 1A). Conversely, 0.1 mg/kg (2.6 μmol/kg per day) did not significantly alter ischemic hind‐limb blood flow compared to PBS control. Thus, the therapeutic dose of 0.5 mg/kg was used for the rest of the study. Figure 1B illustrates the pharmacokinetic profile of plasma H_2_S levels after a single injection of 0.5 mg/kg sodium sulfide, revealing a quick rise of plasma H_2_S levels to ≈32 μmol/L within 1 minute of injection, which falls rapidly back to baseline levels (≈600 nmol/L) within 30 minutes. We next examined steady‐state plasma free H_2_S levels taken 2 hours after the first morning dose of 0.5 mg/kg sodium sulfide, as shown in Figure 1C. Steady‐state free plasma H_2_S is elevated within 1 day of beginning therapy and then decreases between days 3 and 7 and returns to baseline levels by day 10. We next measured tissue free H_2_S levels at days 3 and 7 in nonischemic and ischemic tissues from sodium sulfide– and PBS‐treated mice. Ischemic tissue H_2_S levels from sodium sulfide–treated animals were elevated significantly above those of PBS‐treated controls at day 3 (Figure 1D). Importantly, tissue H_2_S levels were increased only in ischemic tissues, which suggests preferential bioavailability of H_2_S in ischemic tissue. Figure 1E shows that by day 7, tissue H_2_S levels had returned to baseline. Figure 2A through Figure 2D show the representative photomicrographs of angiogenic index in nonischemic and ischemic tissues with or without H_2_S treatment. Figure 2E and Figure 2F represent the H_2_S dose–dependent stimulation of ischemic tissue angiogenesis (CD31/DAPI) and cell proliferation activity (Ki67/DAPI), respectively. These data reveal that H_2_S plays major role in restoration of ischemic hind‐limb blood flow or ischemic vascular remodeling and also indicate rapid and selective augmentation of free H_2_S bioavailability during chronic tissue ischemia.

### H_2_S Restores Ischemic Tissue Blood Flow in a NO‐Dependent Manner

**Figure 3.**
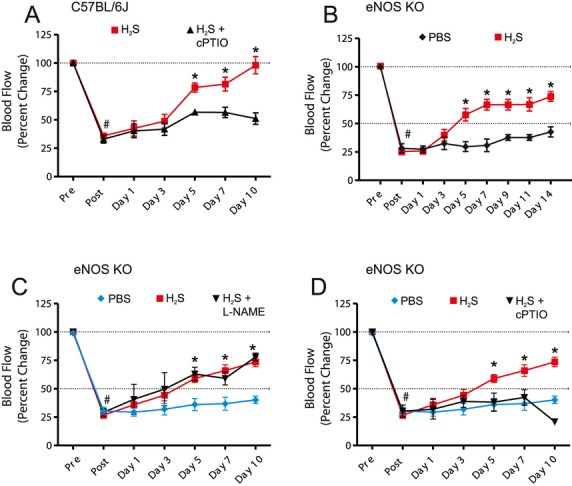
Role of NO and eNOS in H_2_S restoration of ischemic limb blood flow. A, Blood flow from wild‐type animals after sodium sulfide (0.5 mg/kg) therapy with and without cPTIO (1 mg/kg) to scavenge NO. B, Blood flow from eNOS‐knockout (eNOS KO) animals with sodium sulfide therapy (0.5 mg/kg). C, Blood flow results from sodium sulfide–treated eNOS^−/−^ animals plus 5 mg/kg L‐NAME (NOS inhibitor). D, Blood flow results from sodium sulfide–treated eNOS‐knockout animals plus 1 mg/kg cPTIO (NO scavenger). n=12, #*P*<0.05 compared from before to after ligation, *P<0.05 compared to either H_2_S+cPTIO or PBS controls.

**Figure 4.**
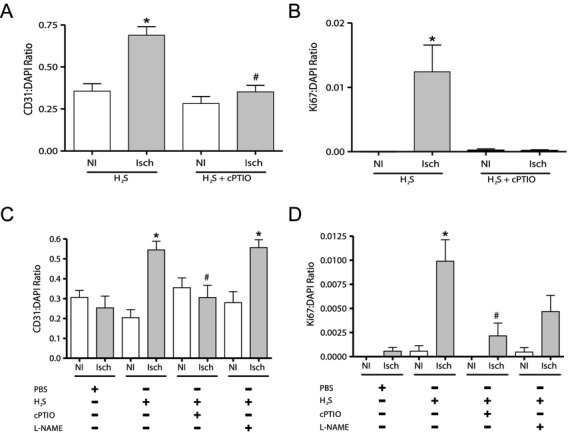
H_2_S enhances vascular density in eNOS^−/−^ mice. A and B, Graphical representation of the CD31:DAPI and Ki67:DAPI ratios demonstrating vascular density and proliferation responses in wild‐type mice treated with cPTIO. C and D, Vascular density and proliferation data of gastrocnemius tissues from eNOS^−/−^ mice under H_2_S, cPTIO, and L‐NAME treatments. NI indicates nonischemic; Isch, ischemic. n=12 animals per cohort, *P<0.05 compared to PBS ischemic tissue data, #*P*<0.05 H_2_S versus H_2_S+inhibitor treatments.

Previous reports suggested that H_2_S confers cardioprotective effects involving NO metabolism.^15,17^ Therefore, we administered an NO scavenger, cPTIO (1 mg/kg), to determine whether NO is involved in mediating ischemic tissue reperfusion during H_2_S therapy. Figure 3A shows that cPTIO significantly blunts the effects of H_2_S therapy on ischemic tissue blood flow in wild‐type mice, demonstrating a key role for NO. Next, experiments were performed with eNOS‐knockout mice to determine whether H_2_S therapy mediates its effects on NO metabolism through this enzyme. Figure 3B shows that H_2_S therapy significantly restored ischemic hind‐limb blood flow in eNOS^−/−^ mice to 75% of preligation levels compared to PBS therapy. Plasma steady‐state H_2_S levels were measured in eNOS^−/−^ mice receiving different treatments: PBS therapy with 0.55±0.06 μmol/L versus sodium sulfide therapy with 0.89±0.06 μmol/L (*P*<0.05) plasma free H_2_S. We next examined whether other NOS activity (inducible NOS [iNOS] and neuronal [nNOS]) could be involved in H_2_S augmentation of eNOS^−/−^ ischemic hind‐limb blood flow by using the NOS blocker L‐NAME (5 mg/kg). L‐NAME did not affect H_2_S‐dependent restoration of eNOS‐deficient ischemic hind‐limb reperfusion, suggesting that other NOS activity is not involved in this response (Figure 3C). Experiments were then performed in eNOS^−/−^ animals with cPTIO plus H_2_S, which completely abrogated H_2_S‐mediated increased ischemic hind‐limb blood flow (Figure 3D). cPTIO completely blocks H_2_S‐mediated angiogenesis and cell proliferation responses in ischemic tissues in wild‐type animals (Figure 4A and Figure 4B, respectively). Importantly, Figure 4C and Figure 4D show that cPTIO but not L‐NAME significantly prevents H_2_S‐mediated increases in the CD31:DAPI and Ki67:DAPI ratios of ischemic tissue in eNOS^−/−^ animals, confirming the importance of alternative NO generation for H_2_S‐mediated ischemic angiogenesis.

### Increased NO Production and cGMP Expression by H_2_S Therapy in Ischemic Tissue

**Figure 5.**
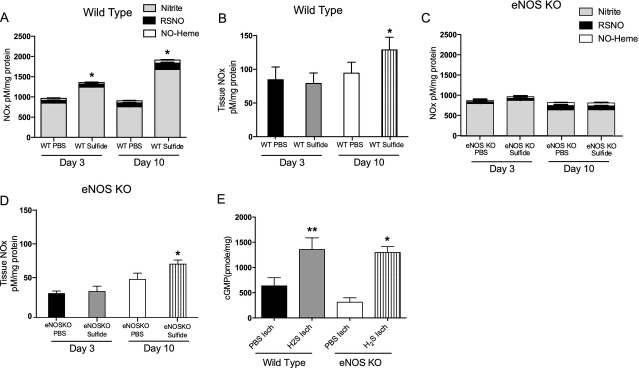
H_2_S effects on plasma and tissue NO formation and bioavailability. A and B, H_2_S‐induced plasma and tissue NOx levels, respectively, in wild‐type mice at day 3 and day 10 after ligation. C and D, H_2_S‐mediated NOx levels in plasma and ischemic tissues of eNOS^−/−^ mice at day 3 and day 10 after ligation. E, H_2_S‐mediated cGMP levels in ischemic and nonischemic tissues from wild‐type and eNOS^−/−^ mice at day 10 after ligation. RSNO indicates nitrosothiol; WT, wild type; KO, knockout; and Isch, ischemic. n=5 animals per cohort. *P<0.05, **P<0.001 compared to PBS control data, #*P*<0.05 compared to eNOS^−/−^ PBS ischemic tissue data.

**Figure 6.**
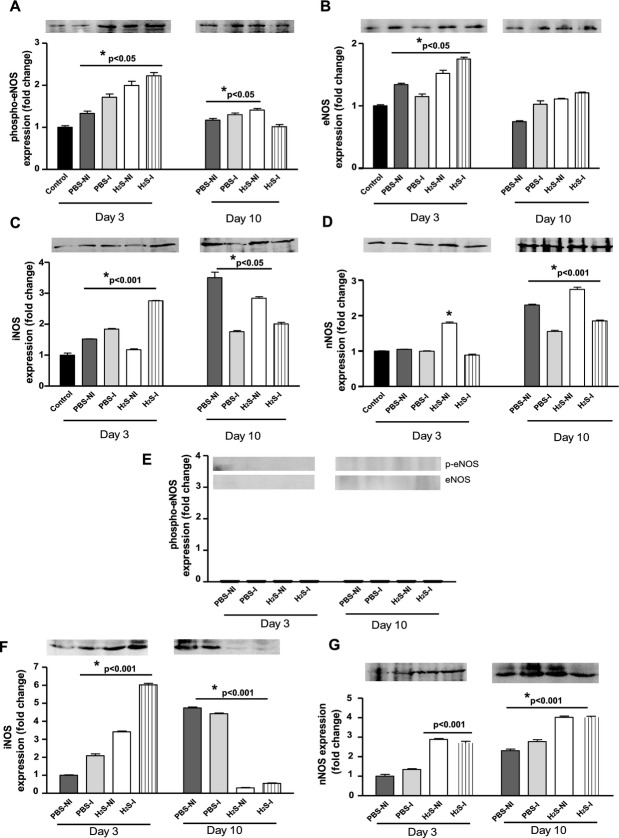
Western blot analyses of NOS isoforms in wild‐type and eNOS^−/−^ mice. Western blots and corresponding quantification below comparing levels of phospho‐eNOS (Ser 1177), total eNOS, iNOS, and nNOS, respectively, from the nonligated (NI) and ligated (I) gastrocnemius muscle tissues of wild‐type mice (A to D) and eNOS^−/−^ mice (E to G), respectively. n=5 animals per genotype per time point, *P<0.05 or *P<0.001 as compared to nonligated tissues. Western blots were repeated 3 times.

Experiments then were performed to measure NO metabolites in plasma and tissue during H_2_S therapy. Figure 5A shows that H_2_S increases plasma nitrite levels at day 3 in wild‐type mice and further increases plasma nitrite, nitrosothiol, and NO‐heme levels by day 10 of therapy. Figure 5B reveals that total NOx levels (a combination of nitrite, nitrosothiol, and nitrosoheme) were selectively and significantly increased in ischemic tissues from wild‐type mice at day 10 alone. Similar NO metabolite measurements were performed in eNOS^−/−^ mice, with Figure 5C illustrating that H_2_S therapy did not significantly increase plasma NO metabolites at either time point. Interestingly, tissue total NOx levels were also selectively increased by H_2_S therapy in ischemic tissues from eNOS^−/−^ mice (Figure 5D), although tissue NOx levels in eNOS^−/−^ mice were smaller than those of wild‐type mice. These findings were confirmed independently with cGMP ELISA, which is indicative of NO generation. Figure 5E shows that H_2_S therapy selectively increases ischemic tissue cGMP production in both wild‐type and eNOS^−/−^ mice. Figure 6 reports Western blot analysis of NOS total protein expression and eNOS phosphorylation at different times in tissues from wild‐type and eNOS^−/−^ mice. These blots reveal that total eNOS expression and phosphorylation was increased at day 3 but not at day 10 in wild‐type mice. Moreover, an increase in total iNOS expression was observed in ischemic tissues at day 3 and decreased by day 10 in both mouse genotypes, whereas nNOS expression was moderately increased in nonischemic and ischemic tissue from wild‐type and eNOS^−/−^ mice. Together, Western blot data, NO metabolite measurements, and ischemic limb blood flow data implicate a role for NOS activity during H_2_S therapy; however, NOS blockade in eNOS^−/−^ mice did not attenuate the effects of H_2_S on ischemic vascular remodeling, suggesting other mechanisms of NO production.

### H_2_S Stimulates Nitrite Reduction to NO Under Hypoxia

**Figure 7.**
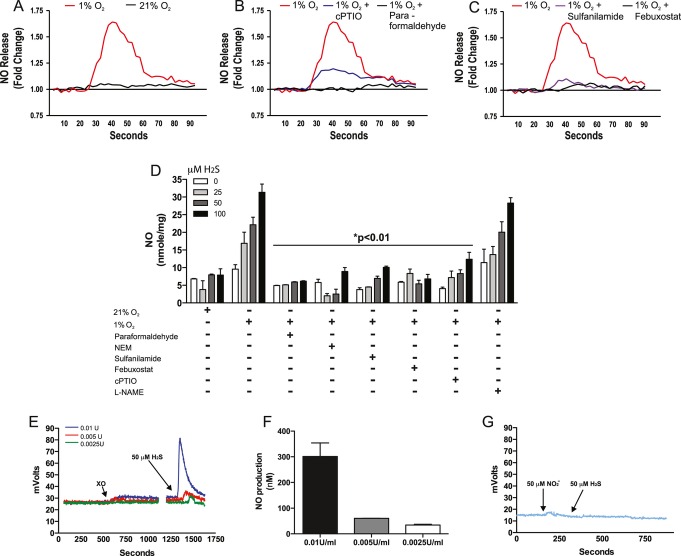
H_2_S stimulates nitrite reduction to NO in hypoxic endothelial cells. A, Release of NO after administration of 50 μmol/L H_2_S with a chemiluminescent NO analyzer under normoxic (21% O_2_) and hypoxic (1% O_2_) conditions. B, Release of NO after administration of 50 μmol/L H_2_S after the addition of cPTIO or paraformaldehyde. C, Release of NO after administration of 50 μmol/L H_2_S after treatment of cells with sulfanilamide or febuxostat. D, Amounts of released NO after administration of different doses of H_2_S in the presence of paraformaldehyde (3%), NEM (10 μmol/L), sulfanilamide (1 mmol/L), febuxostat (10 nmol/L), cPTIO (200 μmol/L), or L‐NAME (300 μmol/L). E through G, Interaction between H_2_S and nitrite followed by amount of NO generation in the presence or absence of recombinant XO (0.0025 U, 0.005 U, and 0.01 U/mg). n=6 replicates performed in triplicate. *P<0.01 compared to PBS control data.

We next examined whether H_2_S could increase NO bioavailability through NOS‐independent mechanisms. Endothelial cells were subjected to normoxic or hypoxic conditions, placed into a NO‐chemiluminescent reaction vessel, and treated with sodium sulfide to examine NO production. Figure 7A shows the release of NO after administration of 50 μmol/L sodium sulfide to either hypoxic or normoxic endothelial cells. H_2_S significantly increased NO release from hypoxic but not normoxic cultured endothelium over a 1‐minute period. Studies were then performed to confirm that the H_2_S‐dependent chemiluminescent peak from hypoxic endothelium was indeed NO. Figure 7B shows that the NO scavenger cPTIO (200 μmol/L) significantly blunted H_2_S‐mediated NO formation. Moreover, protein fixation with 3% paraformaldehyde treatment of hypoxic endothelial cells also prevented H_2_S‐dependent NO formation, indicating that H_2_S‐mediated NO production involves protein function. Figure 7C further demonstrates that H_2_S‐mediated NO formation is nitrite dependent, because sulfanilamide (1 mmol/L) treatment of hypoxic endothelial cells completely prevented NO formation. Treatment with febuxostat (10 nmol/L), a XO inhibitor, blocked H_2_S‐mediated NO formation, implicating XO‐mediated nitrite reduction to NO. Figure 7D reports quantitative measurement of NO formation from dose‐dependent treatment (25 to 100 μmol/L) of H_2_S on hypoxic versus normoxic endothelial cells coupled with various inhibitors of specific molecular targets. Importantly, inhibition of protein thiol modification with NEM, XO with febuxostat, nitrite availability with sulfanilamide, NO bioavailability with cPTIO, or protein fixation with paraformaldehyde all significantly blocked NO production at a range of H_2_S concentrations. In contrast, L‐NAME treatment did not significantly block H_2_S‐mediated NO formation.

To confirm that H_2_S augments XO‐mediated nitrite reduction to NO, we performed recombinant XO protein experiments in conjunction with NO‐chemiluminescent analysis. Figure 7E shows NO‐chemiluminescent traces from the addition of different concentrations of recombinant XO protein to a solution of 50 μmol/L nitrite anion, followed by administration of 50 μmol/L sodium sulfide. Resulting traces reveal a slight increase in NO production upon addition of the highest XO concentration (0.01 U) (as expected) that is followed by a significant spike in NO formation upon addition of sulfide. Figure 7F reports the amounts of NO generation upon addition of 50 μmol/L sodium sulfide to different XO concentrations in the presence of nitrite. Sulfide robustly catalyzes the reaction of 0.01 U of XO to generate ≈300 nmol/L NO from nitrite reduction. Importantly, equimolar (50 μmol/L) addition of sodium sulfide to sodium nitrite in Hank's balanced salt solution alone did not increase NO formation (Figure 7G).

**Figure 8.**
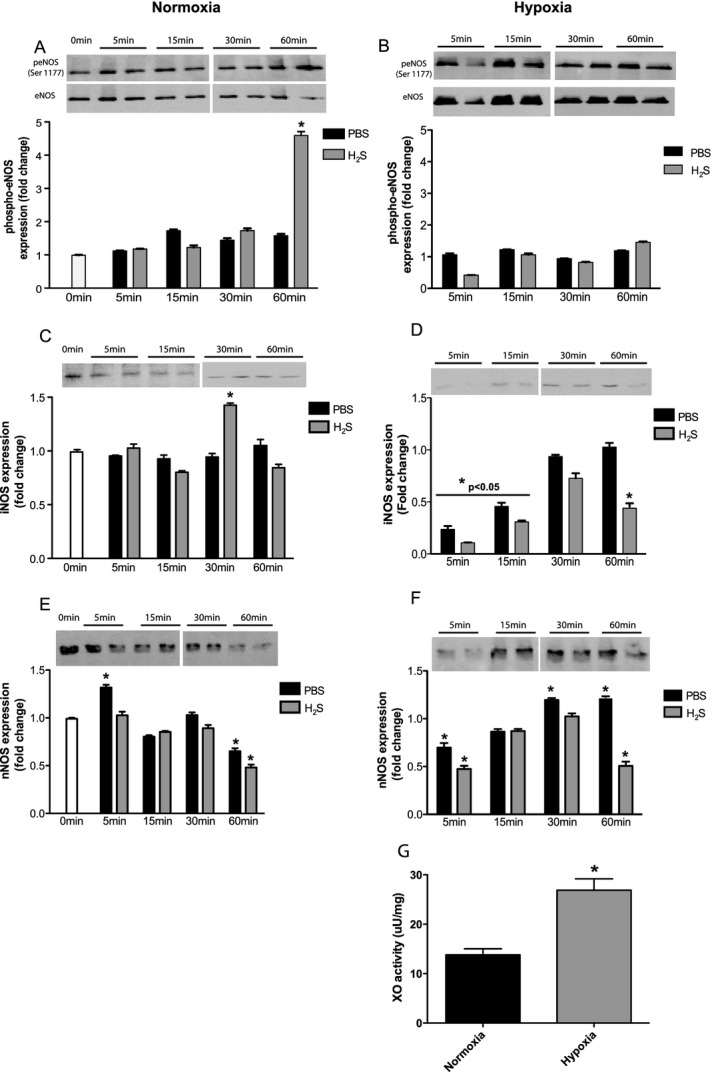
H_2_S effects on eNOS expression and activation in vitro. Mouse endothelial cells were cultured under normoxic or hypoxic conditions and then treated with 50 μmol/L H_2_S and used for NOS Western blot analysis at different time points. A and B, Phospho/total eNOS under normoxia and hypoxia with PBS or sulfide stimulation, respectively. C and D, iNOS expression under normoxia and hypoxia with PBS or sulfide stimulation, respectively. E and F, nNOS expression under normoxia and hypoxia with PBS or sulfide stimulation, respectively. G, Endothelial XO activity under normoxia and hypoxia. n=5, Western blots were repeated in triplicate, *P<0.05 0 min PBS or Normoxia versus v_2_S per time point. Solid line with significance indication illustrates differences between respective time points.

Figure 8 reports the effects of H_2_S on phospho‐eNOS and total eNOS as well as iNOS and nNOS expression in normoxic versus hypoxic endothelial cells. PBS or 50 μmol/L sodium sulfide was added to the cells, which were incubated for 5, 15, 30, and 60 minutes. H_2_S significantly increased phospho‐eNOS levels by 60 minutes, which is consistent with findings from Yusof et al (Figure 8A).^22^ However, H_2_S did not significantly alter eNOS phosphorylation under hypoxic conditions at any time point examined (Figure 8B). Moreover, H_2_S stimulated moderate (≈50%) differences in iNOS expression under normoxic conditions that was not observed under hypoxia (Figure 8C and Figure 8D). Changes in nNOS expression were decreased by H_2_S stimulation under both normoxic and hypoxic conditions (Figure 8E and Figure 8F). Finally, endothelial XO expression and activity was increased under hypoxic conditions, confirming the availability of this key enzyme for H_2_S‐mediated nitrite reduction to NO (Figure 8G). Together, these data clearly demonstrate that H_2_S quickly augments NO formation in hypoxic endothelial cells within minutes after its addition in a XO‐dependent manner and that H_2_S does not significantly increase eNOS expression under hypoxic conditions.

### Increased Xanthine Oxidase Activity Is Required for H_2_S‐Dependent Restoration of Blood Flow in Hind‐Limb Ischemia

**Figure 9.**
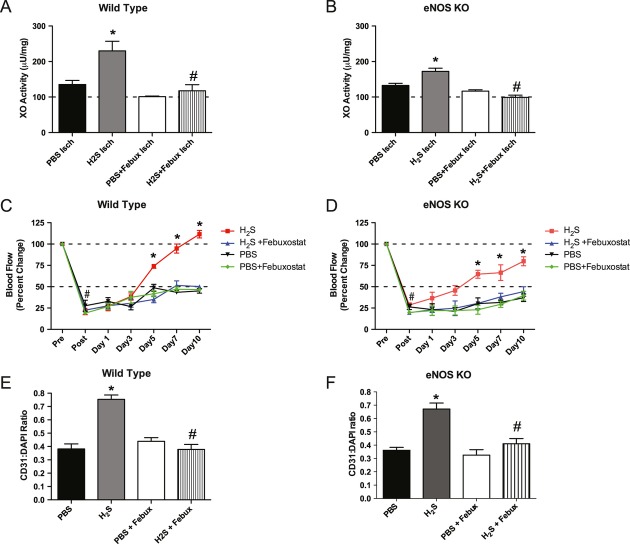
H_2_S increases ischemic tissue XO activity that regulates ischemic tissue reperfusion and vascular remodeling. A and B, XO activity after H_2_S therapy in wild‐type and eNOS^−/−^ mice 10 days after ligation. C and D, Blood flow recovery after febuxostat in wild‐type and eNOS^−/−^ mice in different time points. E and F, Angiogenesis index in wild‐type and eNOS^−/−^ mice 10 days after ligation. n=5 animals, per cohort, *P<0.05 compared to PBS ischemic tissue data and #*P*<0.05 compared to H_2_S ischemic tissue data, or before and after ligation.

Having observed that sulfide can increase XO nitrite reductase activity under hypoxic conditions, we examined whether H_2_S therapy altered XO activity in vivo and whether XO activity was important for H_2_S‐mediated ischemic limb reperfusion. Figure 9A and Figure 9B show that XO activity was significantly increased in H_2_S‐treated ischemic tissues of wild‐type and eNOS‐knockout mice. Treatment with the XO inhibitor febuxostat (5 mg/kg twice a day) blocked H_2_S‐mediated increases in ischemic tissue XO activity, as well as H_2_S‐mediated restoration of ischemic hind‐limb blood flow, in both wild‐type and eNOS^−/−^ mice (Figure 9C and D). Moreover, febuxostat also blunted H_2_S‐mediated increases in angiogenic activity in wild‐type and eNOS^−/−^ mice (Figure 9E and F), demonstrating that XO is required to stimulate the H_2_S‐mediated ischemic angiogenesis.

### H_2_S Increases Ischemic Tissue Hypoxia‐Inducible Factor‐1α VEGF Levels in a NO‐Dependent Manner

**Figure 10.**
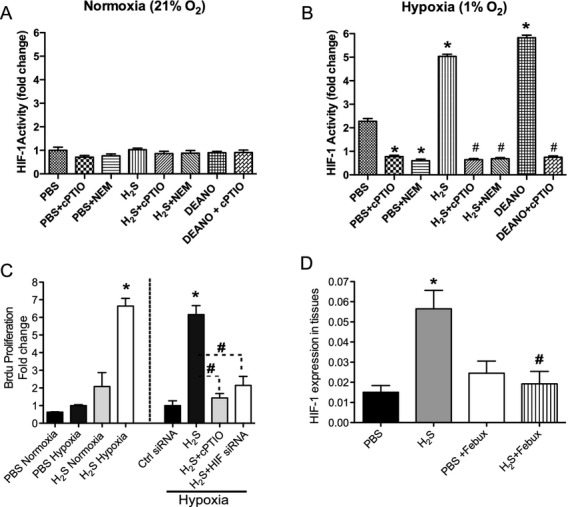
H_2_S stimulates hypoxic endothelial cell proliferation in a NO–HIF‐1α–dependent manner. A, Endothelial bromodeoxyuridine (BrdU) incorporation after H_2_S dose–dependent treatment in normoxic (21% O_2_) conditions. B, Endothelial BrdU incorporation after H_2_S dose–dependent treatment in hypoxic (1% O_2_) conditions. C, Effect of 50 μmol/L sodium sulfide on HIF‐1α activity under normoxic (21% O_2_) conditions. D, Effect of sodium sulfide on HIF‐1α activity under hypoxic (1% O_2_) conditions in vitro. E, Effect of NEM, cPTIO, or HIF‐1α small interfering RNA (siRNA) knockdown on H_2_S‐mediated hypoxic endothelial cell proliferation. n=6 replicates performed in triplicate, *P<0.05 compared to PBS, #*P*<0.05 compared to H_2_S or diethylamine NONOate (DEANO) treatment alone.

**Figure 11.**
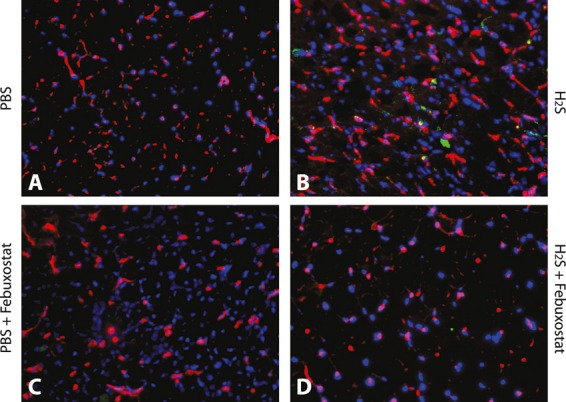
H_2_S increases HIF‐1α expression in ischemic tissues in an XO‐dependent manner. Tissues were stained with anti‐CD31 (red), anti–HIF‐1α (green), and DAPI nuclear counterstain (blue). A, CD31 and HIF‐1α staining in PBS‐treated ischemic tissue at day 10. B, CD31 and HIF‐1α staining in H_2_S‐treated ischemic tissue at day 10. C, CD31 and HIF‐1α staining in PBS+febuxostat treatment. D, CD31α and HIF‐1α staining in H_2_S+febuxostat treatments.

NO and hypoxia‐inducible factor (HIF)‐1α are known to enhance VEGF expression during tissue ischemia.^23,24^ We next examined downstream molecular mechanisms of H_2_S treatment on endothelial cell HIF‐1α activation in an oxygen‐dependent manner. H_2_S treatment of normoxic endothelial cells did not alter HIF‐1α activity over a 12‐hour time period (Figure 10A). However, 50 μmol/L H_2_S stimulated a 5‐fold increase in HIF‐1α activation under hypoxic conditions (Figure 10B). HIF‐1α activation plays an important role in regulating hypoxic endothelial cell proliferation responses and has been shown to be activated by NO.^25,26^ We found that H_2_S stimulated a moderate increase in endothelial cell proliferation at 21% O_2_ (Figure 10C) but that H_2_S elicited a robust 7‐fold increase in endothelial cell proliferation under hypoxia (1% O_2_) that was significantly inhibited by HIF‐1α small interfering RNA or cPTIO. Moreover, we found that H_2_S therapy significantly increased HIF‐1α expression in ischemic tissues, which was completely attenuated by XO inhibition with febuxostat (Figure 10D and Figure 11). Together, these data demonstrate that H_2_S increases HIF‐1α expression and function under low‐oxygen conditions that involve XO activity and NO formation.

**Figure 12.**
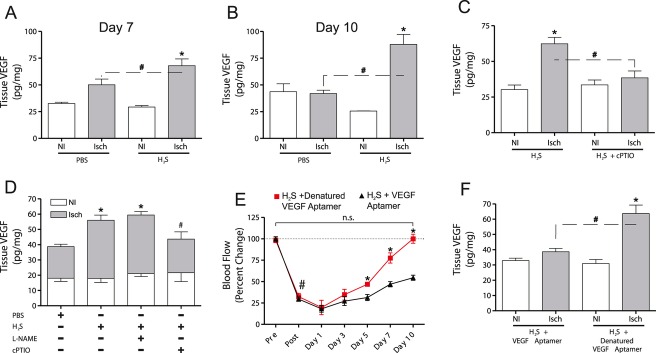
H_2_S increases ischemic tissue VEGF expression that regulates reperfusion. A, Tissue concentrations of VEGF in ischemic and nonischemic tissues of 0.5 mg/kg sodium sulfide–treated animals at 7 days after ligation. B, Tissue concentrations of VEGF in ischemic and nonischemic tissues of sodium sulfide–treated animals at 10 days after ligation. C, Effect of H_2_S+cPTIO on VEGF expression in ischemic and nonischemic tissues. D, Concentrations of VEGF protein in tissues from H_2_S‐treated eNOS^−/−^ mice in the presence and absence of L‐NAME or cPTIO. E, Ischemic limb blood flow values of wild‐type animals treated with H_2_S+VEGF_164_ aptamer or denatured VEGF_164_ aptamer. F, Tissue VEGF levels of wild‐type animals treated with H_2_S+VEGF_164_ aptamer or denatured VEGF_164_ aptamer. n=12 animals per experimental cohort, *P<0.05 compared to nonischemic, or VEGF164 aptamer data; #*P*<0.05 ischemic limb comparison, or before and after ligation.

Next, we examined whether H_2_S augments ischemic tissue angiogenesis and reperfusion involving VEGF production. Figure 12A shows gastrocnemius tissue VEGF levels at day 7 in either PBS‐treated or 0.5 mg/kg H_2_S–treated animals. H_2_S significantly augmented ischemic tissue VEGF levels over that of PBS treatment, as measured by ELISA. H_2_S further increased ischemic tissue levels of VEGF over that of PBS treatment at day 10 (Figure 12B). Importantly, combined treatment of H_2_S plus cPTIO significantly blocked H_2_S induction of VEGF during tissue ischemia, indicating that NO is crucially important for H_2_S augmentation of VEGF during ischemia (Figure 12C). We also discovered that H_2_S could increase ischemic tissue VEGF expression in eNOS‐deficient mice, which was blocked by cPTIO but not L‐NAME (Figure 12D). These data reinforce the importance of H_2_S‐dependent nitrite reduction to NO as a key regulator of ischemic tissue VEGF induction. Lastly, we found that scavenging VEGF with the VEGF_164_ aptamer (Macugen) (50 mg/kg) could significantly prevent H_2_S‐mediated ischemic limb reperfusion in wild‐type animals (Figure 12E). VEGF_164_ aptamer also significantly blunted H_2_S‐mediated VEGF bioavailability in ischemic tissues (Figure 12F). Heat‐denatured VEGF_164_ aptamer (D‐VEGF aptamer) control treatments were unable to block H_2_S‐mediated ischemic limb reperfusion or VEGF expression, revealing that H_2_S selectively stimulates VEGF expression in ischemic tissues in a NO‐dependent manner.

### H_2_S Increases Diabetic Ischemic Hind‐Limb Reperfusion and Angiogenesis in a VEGF‐Dependent Manner

**Figure 13.**
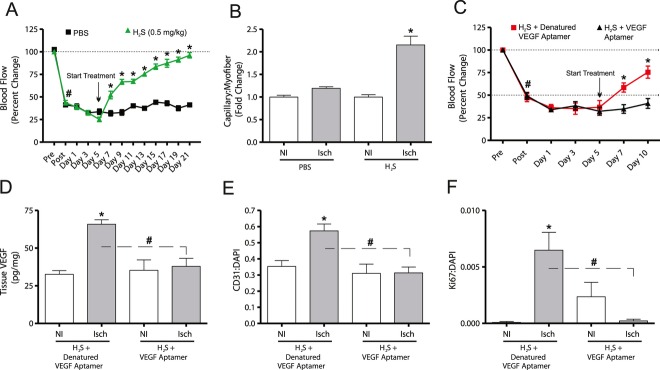
H_2_S restores established diabetic ischemic limb reperfusion and angiogenic activity in a VEGF‐dependent manner. A, Ischemic hind‐limb blood flow changes in 9‐month‐old diabetic mice subjected to femoral artery ligation followed by delayed 0.5 mg/kg sodium sulfide or PBS control therapy. B, Capillary‐to‐myofiber ratio change with sodium sulfide versus PBS therapy between nonischemic and ischemic gastrocnemius muscle tissue. C, Ischemic hind‐limb blood flow in diabetic animals treated with H_2_S+VEGF_164_ aptamer (25 mg/kg IM injection, twice daily) or heat‐denatured VEGF_164_ aptamer. D, Tissue VEGF levels in H_2_S‐treated diabetic animals with VEGF_164_ aptamer or heat‐denatured VEGF_164_ aptamer. E, Graphical representation of the CD31:DAPI ratio demonstrating vascular density in different gastrocnemius tissues from mice treated with H_2_S + denatured VEGF aptamer and H_2_S + VEGF aptamer. F, Graphical representation of Ki67:DAPI ratio demonstrating proliferation in different gastrocnemius tissues from mice treated with H_2_S + denatured VEGF aptamer and H_2_S + VEGF aptamer. For A, B, and C: n=8 animals per experimental cohort, *P≤0.05 compared to PBS control or VEGF_164_ aptamer data; #*P*≤0.05 ischemic limb comparison, or before and after ligation. For D, E, and F: n=8 animals per experimental cohort, *P≤0.05 compared to nonischemic limb H_2_S + denatured VEGF_164_ aptamer data; #*P*≤0.05 ischemic limb comparison.

Impaired ischemic vascular remodeling during diabetes in Db/Db mice involves defective VEGF expression and signaling,^27^ with aged diabetic Db/Db mice manifesting severe defects in ischemic angiogenic responses.^28,29^ We next examined whether exogenous H_2_S therapy could augment ischemic limb reperfusion and vascular growth in 9‐month‐old, aged Db/Db mice subjected to femoral artery ligation. To emulate therapeutic situations, we delayed H_2_S administration until day 5 after ligation, as we have recently reported, thus allowing the establishment of tissue ischemia.^30^ Figure 13A and B illustrate that delayed H_2_S therapy significantly restored ischemic hind‐limb perfusion and capillary‐to‐myofiber ratios compared to PBS control treatment. Figure 13C and D illustrate that cotreatment with H_2_S plus a VEGF_164_‐inhibiting aptamer significantly prevented H_2_S‐mediated ischemic limb reperfusion and increased ischemic tissue VEGF protein levels compared to denatured VEGF_164_ control aptamer treatment. Finally, Figure 13E and F clearly demonstrate that H_2_S plus VEGF_164_ aptamer treatment significantly prevented increased ischemic vascular density and proliferation. Together, these data highlight the potent therapeutic effect of H_2_S on diabetic ischemic vascular remodeling and tissue reperfusion, which depends on increased VEGF expression and angiogenic activity.

## Discussion

H_2_S–NO pathway interactions have been suggested, although specific mechanistic information on how these gasotransmitters influence one another remains poorly understood. Our findings provide significant insight into how H_2_S affects NO metabolism responses during ischemic vascular remodeling such that (1) H_2_S therapy restores ischemic hind‐limb blood flow in a NO‐dependent manner, (2) H_2_S increases NOS expression concomitant with stimulating XO‐mediated nitrite reduction to NO in ischemic tissues, and (3) H_2_S stimulates the ischemic vascular growth by augmenting the expression and activity of HIF‐1α and VEGF in a NO‐dependent manner. We also clearly observed that H_2_S selectively accumulates in and significantly increases NO bioavailability of chronically ischemic tissue. These beneficial effects of H_2_S on ischemic tissue NO levels do not critically depend on NOS activity but do significantly depend on nitrite anion reduction back to NO. These findings further define biochemical mechanisms in which H_2_S increases tissue NO bioavailability that occurs during cardiovascular disease and tissue ischemia.

Exogenous H_2_S has been reported to interfere with NO donor vasodilation responses, presumably through direct interactions generating unknown species.^16^ However, the biological effect of H_2_S seems to be oxygen dependent, as it mediates aortic ring vasoconstriction at high oxygen tensions but vasodilation at low oxygen tension.^31^ Here, we found that H_2_S treatment increased ischemic vascular remodeling, consistent with previous studies; however, our results identified H_2_S‐dependent release of NO through nitrite reduction as a key cytoprotective mechanism during tissue ischemia.^32^ Previous studies from Cai et al and Papapetropoulos et al did not observe H_2_S‐mediated NO formation in their angiogenesis studies.^32,33^ Conversely, Coletta and coworkers recently have reported that mutual interaction between H_2_S and NO might be important for physiological control of vascular function.^34^ Specifically, they reported that H_2_S modulates wound healing responses, vasodilation, and vessel sprouting, involving activation of PI3K/Akt/phospho‐eNOS and NO bioavailability. However, no studies have examined molecular mechanisms of H_2_S on NO metabolism under chronic ischemia/hypoxic conditions. Previously, Wang et al reported that H_2_S therapy during tissue ischemia did not alter plasma NO levels^35^; however, this study only examined plasma nitrite/nitrate levels with the Griess reaction methodology, which is unable to detect physiological changes in plasma or tissue NO metabolites (eg, NO, nitrosothiols, nitrosoheme). Using the NO‐chemiluminescent measurement technique, we clearly observed multiple effects of exogenous H_2_S on NO formation involving different mechanisms and metabolite species. It has been suggested that some of the beneficial cardiovascular effects of H_2_S could involve eNOS activation and expression.^15,17^ However, our data indicate that eNOS expression or function is not essential for H_2_S‐mediated ischemic vascular remodeling or NO formation, revealing that H_2_S stimulation of XO‐dependent nitrite reduction to NO acts as an alternative mechanism to maintain NO bioavailability. Our findings are consistent with classical studies from the Massey laboratory that demonstrated the importance of hydropersulfide formation at the active site of XO in regulating its activity, such that sodium sulfide was shown to increase XO protein activity, which we confirmed in vivo and in vitro.^36,37^

**Figure 14.**
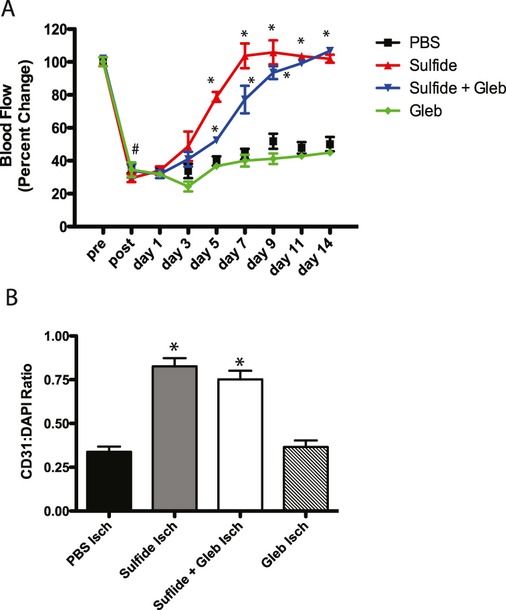
Inhibition of K‐ATP channels on H_2_S‐mediated ischemic vascular remodeling. A, Effect of glibenclamide (1.4 mg/kg)–mediated K‐ATP channel inhibition on H_2_S‐dependent ischemic limb reperfusion. B, Effect of glibenclamide plus H_2_S on ischemic vascular CD31:DAPI density. n=8 animals per treatment cohort, *P<0.05 versus PBS ischemia.

We and others have reported that nitrite anion reduction to NO mediates cytoprotection during acute ischemia‐reperfusion injury and chronic tissue ischemia.^19,38^ We previously have reported that nitrite‐dependent NO formation rapidly augments ischemic hind‐limb reperfusion and ischemic vascular remodeling.^19^ The biological potency of H_2_S treatment for chronic tissue ischemia is similar to that of sodium nitrite, which suggests that a major pathway of the beneficial effects of H_2_S might occur via nitrite‐dependent NO formation apart from other previously defined effects, such as antioxidant, signaling (K‐ATP or phosphodiesterase 5), and transcription factor changes. Figure 14 supports this hypothesis, because blockade of K‐ATP channels (known to be activated by H_2_S) with glibenclamide does not prevent H_2_S‐increased ischemic hind‐limb blood flow or ischemic vascular remodeling. Taken together, it appears that XO‐mediated nitrite reduction to NO plays a key role in mediating the beneficial effects of H_2_S for ischemic vascular remodeling.

NO formation through XO‐mediated nitrite reduction critically serves to maintain NO bioavailability in addition to NOS‐dependent mechanisms.^39^ Thus, XO is an important source for NOS‐independent NO generation under conditions in which NOS activity could be limiting, and our findings clearly show that sulfide can stimulate recombinant XO‐dependent nitrite reduction to NO. This most likely occurs through posttranslational modification of XO (presumably sulfhydration) that is currently being studied in greater detail. Nonetheless, in vitro and in vivo XO‐inhibition studies revealed XO activation to be a dominant mediator of ischemic tissue NO formation because febuxostat, but not L‐NAME inhibition of NOS activity, completely prevented the beneficial effects of H_2_S in eNOS^−/−^ mice. This is confirmed by the observation that XO‐mediated H_2_S‐induced NO production can enhance cGMP production in ischemic tissue of eNOS^−/−^ and wild‐type mice. Future studies will further examine how H_2_S affects XO activity in vivo and in vitro regulating NO production and bioavailability.

Recent reports suggest that H_2_S could positively regulate HIF‐1α activity.^40,41^ These studies showed that H_2_S‐enhanced hypoxia induced HIF‐1α and VEGF expression in vascular smooth muscle cells, which enhanced endothelial cell viability. Our findings further advance our understanding of H_2_S and HIF‐1α because H_2_S stimulation of endothelial cell proliferation was maximal under hypoxia that critically required NO‐dependent HIF‐1α activity. Endogenous NO is known to stimulate VEGF synthesis and VEGF‐induced angiogenesis that is blunted by NOS inhibition.^42–44^ Likewise, angiogenesis is impaired when NO bioavailability is attenuated, as seen during hind‐limb ischemia in eNOS‐knockout mice that is not significantly reversed by administration of VEGF.^45^ Moreover, NO along with cGMP production exerts these effects by increasing expression of VEGF.^42,46,47^ Our results are in agreement with these previous reports and reveal that H_2_S increases VEGF expression in eNOS^−/−^ ischemic tissues in a NO‐dependent manner that can stimulate angiogenesis in ischemic tissues. The implications of our results are striking, as ischemic VEGF induction in eNOS^−/−^ mice is ultimately NO dependent, which reinforces the importance of crosstalk between H_2_S and NO metabolism pathways.

In conclusion, we have discovered that H_2_S selectively augments NO production and associated downstream vascular growth and remodeling in chronically ischemic tissues by influencing NOS expression and critically stimulating nitrite reduction to NO via XO. H_2_S has been proposed to be a novel therapeutic for ischemia‐reperfusion injury; however, our data also demonstrate its benefit for NO‐dependent ischemic vascular disorders that have yet to achieve broad clinical resolution.^48^ Further studies are needed to better understand the biological and therapeutic implications of H_2_S‐dependent nitrite reduction to NO, including downstream signaling pathways that are activated to confer ischemic tissue cytoprotection.
